# *Drosophila* comet assay: insights, uses, and future perspectives

**DOI:** 10.3389/fgene.2014.00304

**Published:** 2014-08-29

**Authors:** Isabel Gaivão, L. María Sierra

**Affiliations:** ^1^Department of Genetics and Biotechnology, Animal and Veterinary Research Centre, University of Trás-os-Montes and Alto DouroVila Real, Portugal; ^2^Área de Genética, Departamento de Biología Funcional, and Instituto Universitario de Oncología del Principado de Asturias, Universidad de OviedoOviedo, Spain

**Keywords:** *Drosophila*, comet assay, neuroblast cells, hemocytes, midgut cells, genotoxicity, DNA repair

## Abstract

The comet assay, a very useful tool in genotoxicity and DNA repair testing, is being applied to *Drosophila melanogaster* since around 15 years ago, by several research groups. This organism is a valuable model for all kind of processes related to human health, including DNA damage response. The assay has been performed mainly *in vivo* using different larvae cell types (from brain, midgut, hemolymph, and imaginal disk), but also *in vitro* with the S2 cell line. Since its first application, it has been used to analyze the genotoxicity and action mechanisms of different chemicals, demonstrating good sensitivity and proving its usefulness. Moreover, it is the only assay that can be used to analyze DNA repair in somatic cells *in vivo*, comparing the effects of chemicals in different repair strains, and to quantitate repair activities *in vitro*. Additionally, the comet assay in *Drosophila*, *in vivo* and *in vitro*, has been applied to study the influence of protein overexpression on genome integrity and degradation. Although the assay is well established, it could benefit from some research to determine optimal experimental design to standardize it, and then to allow comparisons among laboratories independently of the chosen cell type.

## INTRODUCTION

The single cell gel electrophoresis test, or comet assay, was originally developed by Östling and Johanson (1984) as a micro-electrophoretic technique to visualize DNA damage in single cells. Subsequently it was improved by Singh et al. (1988), and since then so extensively used that some working-groups were created to standardize its application to mammal and human cells studies (Burlinson et al., 2007; Karlsson, 2010; Azqueta and Collins, 2013; Ersson et al., 2013; Godschalk et al., 2013; Collins et al., 2014).

Its usefulness and easy performance lead to its rapid application to several fields, like genotoxicity analyses (Speit and Hartmann, 1999; Tice et al., 2000; Hartmann et al., 2003; Collins, 2004), human population biomonitoring (Collins et al., 1998; Somorovská et al., 1999; Kassie et al., 2000; Møller et al., 2000; Faust et al., 2004; Hoffmann et al., 2005; Burlinson et al., 2007; Dusinska and Collins, 2008; Uriol et al., 2013) and DNA repair (Collins and Horváthová, 2001; Collins et al., 2001; Collins and Gaivão, 2007; Gaivão et al., 2009; Dusinska and Collins, 2010). Because of this, it was also applied to other organisms, using different cell types (Menke et al., 2001; Dixon et al., 2002; Lee and Steinert, 2003; Jha, 2008; Dhawan et al., 2009; Ventura et al., 2013).

Surprisingly, its application to *Drosophila melanogaster* was rather late, despite the fact that this organism is one of the most valuable higher eukaryotic model organism, for all kind of processes and situations related to human health (Reiter et al., 2001; Koh et al., 2006; Wolf et al., 2006; Khurana et al., 2006; Rand, 2010), including the *in vivo* DNA damage response processes (Søndergaard, 1993; Vogel et al., 1999; Sekelsky et al., 2000; Vecchio, 2014). The first attempt to apply the comet assay to *Drosophila in vivo* was performed by Gaivão (1999) in her Ph.D. Thesis, checking the availability of imaginal disk and brain ganglia cells. In the first published work, appeared 3 years later, the comet assay was performed with brain ganglia cells from third instar larvae (Bilbao et al., 2002). As with other organisms, several cell types, apart from the brain cells, have been used to carry out this assay in *Drosophila in vivo*, such as midgut cells (Mukhopadhyay et al., 2004; Siddique et al., 2005a; Sharma et al., 2011), hemocytes (Carmona et al., 2011a), and imaginal disk cells (Verma et al., 2012).

Most of these authors used the comet assay for its original purpose, the *in vivo* analyses of genotoxicity and DNA repair. But more recently, this assay has also been used to study genotoxicity *in vitro* (Guanggang et al., 2013), to analyze the influence of protein overexpression on genome integrity *in vivo* (Plyusnina et al., 2011; Brennan et al., 2012; Verma et al., 2012) and *in vitro* (Radyuk et al., 2006), and very recently to quantitate DNA repair activity *in vitro* (Rodríguez et al., submitted).

In this mini-review we aim to present available information about the comet assay in *Drosophila*; outlining the type of cells and insights into its technical performance, its uses *in vivo* and *in vitro*, and its spread availability as a useful tool and future perspectives.

## INSIGHTS

### BRAIN CELLS

The *Drosophila* comet assay using brain ganglia cells was developed at the University of Oviedo (Spain) by Isabel Gaivão and the group of L. María Sierra and M. A. Comendador (Gaivão, 1999; Bilbao et al., 2002). Our aim was to develop a tool to study both genotoxicity and *in vivo* DNA repair in somatic cells.

The developed protocol included the use of third instar larvae (developed 24 h at 24°C and five additional days at 21°C) treated in the food during 12 h. Brain ganglia were extracted, and cells were mechanically individualized, shredding the tissue with tungsten wires, and suspended in Ringer’s buffer (Bilbao et al., 2002; García-Sar et al., 2008, 2012; Rodríguez et al., submitted). Cells were embedded in 0.5% low melting point agarose (LMPA), three agarose layers were prepared, and cells were disrupted during 2 h with a lysis solution containing *N*-lauroylsarcosine sodium salt (*N*-LS), 0.77%, and dimethyl sulfoxide (DMSO) 10%. Denaturation was performed at pH 12.6, for 20 min, and electrophoresis was set at 0.9 V/cm, for 20 min. After neutralization and fixation, slides were stained with ethidium bromide (0.4 μg/mL), with Vectashield^®^ fluorescence protector (Vector Laboratories, Inc., Burlingame, CA, USA) to avoid fluorescence decay (**Table 1**). A very detailed protocol was recently published (Sierra et al., 2014).

**Table 1:** Methodological details, analyzed agents, and results of genotoxicity and/or DNA repair studies carried out in *D. melanogaster* with the comet assay.

Cell type	Treat. time (h)	%Agarose	Denaturat pH/time (min)	Electrophoresis V/cm/time (min)	Staining	Strain	Agents	Results	Reference
Brain cells	12	0.5	12.6/20	0.9/20	EthBr 40 μL (0.4 μg/mL)	*OregonK, mus201*	cDDP	+	García-Sar et al. (2008, 2012)
						*OregonK, mus201, mus308,double mut*	MMS	+	Rodríguez et al. (submitted)
			10–12.6/20			*OregonK, mus201, mus308*	MMS	+	Bilbao et al. (2002)
							EMS	+	
							ENU	+	

Hemocytes	24 ± 2	0.75	13/25	0.7/20	DAPI 20 μL (1 μg/mL)	*OregonR*	EMS	+	Carmona et al. (2011a)
							Cr(VI)-K_2_Cr_2_O_7_	+	
							γ-rays	+	
							PbCl_2_	-	Carmona et al. (2011b)
							Pb(NO_3_)_2_	+	
							NiCl_2_	-	Carmona et al. (2011c)
							NiSO_4_	+	
			13/20	0.73/25			AuNP	+	Sabella et al. (2011)

Mid-gut cells	74	0.75	> 13/10	0.7/15	EthBr 75 μL (20 μg/mL)/ 10 min	*OregonR*	Cypermethrin	+	Mukhopadhyay et al. (2004)
							Industrial waste leachates	+	Siddique et al. (2005b)
	24					*OregonR*	EMS	+	Siddique et al. (2005a)
							MMS	+	
							ENU	+	
							CP	+	
						*OregonR* plus FPG and EndoIII enzimes	H_2_O_2_	+	Shukla et al. (2011)
							CdCl_2_	+	
							CuSO_4_	+	

	24/48					Transgenic (*hsp70-lacZ)Bg*	Graphene-Cu_2_O nanocomposite	+	Siddique et al. (2013)
	48					*OregonR, mei9, mus201, mus210, mei41, mus207, mus209, mus309*	Cr(III)-CrCl_3_	-	Mishra et al. (2011)
							Cr(VI)-K_2_Cr_2_O_7_	+	
							Dichlorvos	+	Mishra et al. (2014)
	12–48					*OregonR* transgenic *hsp70, hsp83, hsp26* plus FPG and EndoIII enzimes	Endosulfan	+	Sharma et al. (2012)
	48/72					*OregonR, mei41, mus201, mus308, rad54*	Industrial waste leachates	+	Siddique et al. (2008)
	48		8.5/60	14V-100mA/60		*OregonR*	CP	+	Sharma et al. (2011)
							BLM	+	
							cDDP	+	
							Cr(VI)	+	
	48/24		8.5/60	14V-60mA/60		*OregonR, ligiV, ku80, spn-A, okr, mre11*	Cr(VI)-K_2_Cr_2_O_7_	+	Mishra et al. (2013)

Imaginal disk	48	0.75			Propidium iodide (1 μg/mL)	*OregonR, Act-GAL4/CyO;*+/+*, Act-GAL4/Pol𝜖RNAi;*+/+*,*+/+*;pl10R/pl10R*	BLM	+	Verma et al. (2012)

Cultured cells	6/24	0.5	Alkal/30	1/10	SYBR green	*S2* cell line: standard and transfected	Paraquat	+	Radyuk et al. (2006)
							*S*-nitroso-*N*-acetyl penicillamine	+	
	24/48	1	13/10	1/10	EthBr 40 μL (20 μg/mL)	*S2* cell line	Methomil	+	Guanggang et al. (2013)

Microscope photos were analyzed with the Komet 5 software program (Kinetic, England), collecting data on % tail DNA, tail length, and tail moment, although the analyses were carried out with the tail moment parameter because it increased linearly with the amount of DNA damage and was the best to detect statistically significant differences. The wild-type OregonK *Drosophila* strain was used as a standard, since it is rather sensitive to the action of DNA damaging agents in somatic cells (Gaivão and Comendador, 1996). Under all these conditions, the comet assay yielded spontaneous DNA damage measurements of 6.5 ± 0.5 for tail moment and of 30 ± 1.25 for %tail DNA.

Recently, we have developed a technical variation of this protocol to be able to quantitate DNA repair activities *in vitro*. This variant consists on the incubation of nucleoid DNA with cell-free protein extracts from repair-efficient and deficient-strains, after the lysis step (Rodríguez et al., submitted).

Plyusnina et al. (2011) also used brain cells to perform the comet assay. They disaggregated them mechanically in Poels’ salt solution (PSS). Cells were embedded in 0.75% LMPA, lysis was performed for 1 h, with a buffer without *N*-LS or DMSO. Denaturation was carried out at pH 13 for 10 min, followed by electrophoresis at 15 V–300 mA for 10 min. Nuclei were stained with acridine orange. Comet images were analyzed with the Comet Score^TM^ software (TriTek Corporation, USA), and the parameter for analysis was the Olive tail moment. The wild-type strain was Canton-S and the values of spontaneous DNA damage measurements were approximately 1.2 units of the analyzed parameter.

### HEMOCYTES

The comet assay using hemocytes from *Drosophila* was developed by the group of R. Marcos at the Autonomous University of Barcelona (Spain). In this protocol, 72 ± 2 h old larvae (developed at 24°C) were treated for 24 h. Since hemocytes are individual cells, they were just collected in phosphate buffered saline (PBS), with 0.07% phenylthiourea (Carmona et al., 2011a,b,c; Sabella et al., 2011).

Cells were embedded in 0.75% LMPA, and two agarose layers were prepared. Lysis buffer contained *N*-LS 1% (Carmona et al., 2011a,b,c), or DMSO 10% (Sabella et al., 2011). Lysis time was 2 h. Small variations on the denaturation time and the electrophoresis conditions were performed (**Table 1**). Nucleoids were stained with DAPI (1 μg/mL). Detailed protocols for this assay are available (Marcos and Carmona, 2013; Sierra et al., 2014).

Comets were analyzed with the Komet 5 software program, and results were mostly expressed as % tail DNA (Carmona et al., 2011a,b,c), although DNA damage was also measured as percentage of damaged nuclei (Sabella et al., 2011). The standard wild-type strain used was OregonR, an insecticide resistant strain with high levels of cytochrome P450 and xenobiotic metabolism (Hällström et al., 1984). With this protocol, the highest % tail DNA detected for spontaneous DNA damage was 18.93 ± 0.84 (Carmona et al., 2011c).

### MIDGUT CELLS

The comet assay with midgut cells was developed by the group of A. Dhawan and D. K. Chowdhuri at the CSIR-Indian Institute of Toxicology Research, formerly Industrial Toxicology Research Center (India). They also developed the enzymatic brain cell disaggregation protocol. Mid-gut tissue, with or without brain ganglia, from third instar larvae treated for different times were explanted in PSS buffer. Cells were enzymatically individualized, incubating 15 min with collagenase (0.5 mg/mL) in PBS. Treatment times varied from 12 to 74 h (**Table 1**; Mukhopadhyay et al., 2004; Siddique et al., 2005a,b, 2008, 2013; Mishra et al., 2011, 2013, 2014; Sharma et al., 2011, 2012; Shukla et al., 2011).

Cells were embedded in 0.75% LMPA, with two or three agarose layers. Lysis buffer did not contained *N*-LS, or DMSO, and lysis time was 2 h. As presented in **Table 1**, the denaturation step was mainly performed at pH > 13 during 10 min, although in two works this step was performed at neutral conditions, pH 8.5 for 60 min (Sharma et al., 2011; Mishra et al., 2013). In these two cases electrophoresis was also set up differently from the more standard 0.7 V/cm during 15 min (**Table 1**). Staining was carried out with ethidium bromide (20 μg/mL), for 10 min.

Some of the works carried out at the CSIR-Indian Institute of Toxicology Research analyzed three comet parameters, % tail DNA, tail length, and Olive tail moment (Mukhopadhyay et al., 2004; Siddique et al., 2005a,b), and in others only the % tail DNA was used for result analyses. The Komet 5 software program was throughout used for photo analysis, except by Siddique et al. (2013), who used the Comet Score^TM^ software, v1.5, to analyze tail length. The standard wild-type strain was OregonR. With this protocol, % tail DNA varied from 6 to 10%, with errors lower than 1%, and Olive tail moment varied from 0.7 to 1.5, with errors under 0.12.

### IMAGINAL DISK CELLS

Imaginal disk cells have also been used to carry out the comet assay *in vivo* in *Drosophila* (Verma et al., 2012). In this case, cell disaggregation was performed enzymatically, as described earlier for midgut cells (see Midgut Cells). The conditions of the comet assay were also those described above (see Midgut Cells) with two exceptions: the lysis buffer contained DMSO 10%, and nuclei were stained with propidium iodide (1 μg/mL). Photos were analyzed with the Comet Score^TM^ software, and DNA damage was quantified using the % tail DNA parameter. The wild-type strain used was OregonR, and the spontaneous values of % tail DNA were around 7 (only a graph was presented).

### OTHER CELLS

Spermatocytes were other cell type chosen to perform the comet assay *in vivo*, in this case from *D. simulans* (Brennan et al., 2012). Testes were dissected in PBS. However, with respect to the comet assay, the only information available from this work is that they have used the OxiSelect Comet Assay Kit (from Cell BioLabs, San Diego, CA, USA) to perform it, the Comet Score^TM^ software for image analysis, and a classification of % tail DNA in five categories for the analysis of results.

The comet assay in *Drosophila* was also performed *in vitro* using S2 cultured cells (Radyuk et al., 2006; Guanggang et al., 2013). Cells were treated for 24 h, embedded in 0.5% LMPA, lysed for 30 min, denatured in alkaline conditions for 30 min, electrophoresed at 1 V/cm for 10 min, and stained with SYBR green dye; and the DNA damage was measured classifying the damaged cells in four categories (Radyuk et al., 2006).

Alternatively, cells were treated for 24 or 48 h and embedded in 1% LMPA. Lysis buffer contained DMSO 10%, and lysis time was 30 min. Denaturation at pH 13 for 10 min was followed by electrophoresis 1 V/cm for 10 min. Nucleoids were stained with ethidium bromide (20 μg/mL), and comet photos were analyzed with CASP image analysis system, measuring % tail DNA and tail moment. The values of these parameters for spontaneous DNA damage were 11.57 ± 5.84 for % tail DNA and 2.20 ± 1.24 for tail moment (Guanggang et al., 2013).

## USES

### GENOTOXICITY AND DNA REPAIR ANALYSIS

It is possible to study DNA repair *in vivo* in *Drosophila* germ cells, male and female ones, since many years ago (Vogel et al., 1996; Hernando et al., 2004). However, it was not possible to study it in somatic cells, with the available *in vivo* SMART assays (Vogel and Nivard, 2001). Because of this, our main aim when designing the first comet assay protocol in *Drosophila* was to develop a tool to study DNA repair *in vivo* in somatic cells (Gaivão, 1999; Bilbao et al., 2002). Consequently, many (but not all) of the works carried out with this assay in *Drosophila* were aimed to study genotoxicity and/or DNA repair in somatic cells *in vivo*.

In addition to its use in the assay design, using model genotoxic agents, and efficient and deficient repair strains (Bilbao et al., 2002), brain cells, obtained with the University of Oviedo protocol, were used to demonstrate the relationship between cisplatin induced adducts and DNA strand breaks (García-Sar et al., 2008), and the influence of the nucleotide excision repair system in this relationship, with the *in vivo* comet repair assay (García-Sar et al., 2012). Very recently, brain cells have been used to implement the *in vitro* comet repair assay in *Drosophila*, to be able to quantitate DNA repair activities *in vitro* (Gaivão et al., 2014), and it was used to check the repair activity of cell free protein extracts obtained from wild-type and repair mutant strains in the repair of methyl methanesulfonate induced DNA damage (Rodríguez et al., submitted).

After checking their use with known inducers of DNA strand breaks (Carmona et al., 2011a), hemocytes were used to demonstrate that not all the salts of lead and nickel were genotoxic (Carmona et al., 2011b,c), but that gold nanoparticles were so (Sabella et al., 2011).

Midgut cells, with or without brain cells, have been used to study oxidative DNA damage, using incubations with FPG and Endo III enzymes (Shukla et al., 2011; Sharma et al., 2012), and to demonstrate the genotoxicity of chromium salts (Mishra et al., 2011, 2013; Sharma et al., 2011), pesticides like cypermethrin (Mukhopadhyay et al., 2004), endosulfan (Sharma et al., 2012), and dichlorvos (Mishra et al., 2014), contaminants as industrial waste leachates (Siddique et al., 2005b, 2008), and nanomaterials like graphene copper nanocomposite (Siddique et al., 2013). In addition, some of these genotoxic agents, like chromium salts, dichlorvos, and industrial waste leachates, were analyzed in different repair conditions, with the *in vivo* comet repair assay (Siddique et al., 2008; Mishra et al., 2011, 2013, 2014), checking the influence of pre- and post-replication DNA repair pathways on their genotoxicity. Other genotoxic agents, like endosulfan and graphene copper nanocomposite, were analyzed in transgenic strains for genes encoding heat shock proteins (hsp), to check responses to xenobiotic stress, and influence of xenobiotic metabolism (Sharma et al., 2012; Siddique et al., 2013).

Analysis of genotoxicity, specifically that of the insecticide methomil, was also the aim of the comet assay performed *in vitro* with S2 culture cells (Guanggang et al., 2013).

### OTHER USES

In addition to these studies of genotoxicity and DNA repair, the comet assay *in vivo* in *Drosophila* had been used to study: (i) the influence of GADD45 protein over-expression on longevity and spontaneous DNA damage, as an indication of increased DNA repair activity (Plyusnina et al., 2011); (ii) chromatin integrity in DNA polε mutants exposed to bleomycin (Verma et al., 2012); and (iii) oxidative DNA damage in spermatocytes of *Wolbachia*-infected *D. simulans* flies (Brennan et al., 2012).

Furthermore, the comet assay *in vitro* was used to check the effect of mitochondria ectopic over-expression of dOgg1 and RpS3 proteins on DNA degradation after oxidative damage induction (Radyuk et al., 2006).

## FUTURE PERSPECTIVES

Considering the relevance of *D. melanogaster* as an established insect model for human diseases and toxicological research, recommended by the European Centre for the Validation of Alternative Methods (ECVAM), all the results of *in vivo* genotoxicity studies with this organism should be considered as relevant for human health. In this aspect, the comet assay performed *in vivo* is even more important because, in addition to its high sensitivity, it is the only assay that allows the analysis of DNA repair in somatic cells. And, at least theoretically, the comet assay results should be more easily and directly compared among species.

There is however a possible problem: there are several groups using different protocols, what make comparisons even among *Drosophila* laboratories impossible. So, it is necessary to standardize the basic comet assay protocol. Azqueta et al. (2011) demonstrated in human cells how small changes in some variables, such as agarose concentration, alkaline unwinding time, or electrophoresis conditions, might significantly affect the results. And these are specifically some of the variables that differ between the protocol for brain cells and the rest: LMPA percentage (0.5 vs. 0.75%), lysis buffer composition (*N*-LS and DMSO vs. only *N*-LS or none of them), or denaturation and electrophoresis conditions (more V/cm, compared to the protocol for hemocytes, and more denaturation time and V/cm, compared to the protocol for midgut cells). These differences might explain the higher values of the comet parameters, for spontaneous DNA damage, found with the brain cell protocol, compared to the others, because although some differences might be attributed to the wild-type strain analyzed (OregonK is more sensitive than OregonR), at least in the case of human cells differences due to individuals or cell types were not so relevant (Azqueta et al., 2011). It is then necessary to study the effects of these differences and whether a higher sensitivity is an advantage or a disadvantage.

To help with the required standardization, some of the protocol optimizations performed for other cells and organisms can be tested and applied to *Drosophila*, including its simplification (number of layers, size of gels, or solution compositions) and the high throughput versions, recently developed based on the use of 12 mini-gels on one slide (Shaposhnikov et al., 2010). Additionally, the modified comet assay performed incubating with repair lesion-specific enzymes, as used by Shukla et al. (2011) and Sharma et al. (2012) for oxidative damage, can be extended to other types of damages and repair systems (Collins et al., 2008). This standardization would also clearly help the use of this assay in other types of studies, different from genotoxicity and DNA repair testing.

## Conflict of Interest Statement

The authors declare that the research was conducted in the absence of any commercial or financial relationships that could be construed as a potential conflict of interest.
